# HBZ RNA may promote cell survival against clastogenic damages and thereby contributes to the development of leukemic ATL cells

**DOI:** 10.1186/1742-4690-11-S1-O56

**Published:** 2014-01-07

**Authors:** Marco Goicochea, Toshie Nata, Robert Gallo, Hua Cheng, Yutaka Tagaya

**Affiliations:** 1 Institute of Human Virology, University of Maryland School of Medicine, Baltimore, MD, USA

## 

HTLV-1, the first tumorigenic retrovirus identified in humans, causes adult-T cell leukemia in about 5% of the infected individuals with a long latency period > 40 years. The mechanism of ATL has been extensively studied with emphasis on the role of regulatory factors encoded by the viral genome including Tax, Rex, p30 p12, p8 and p13. However, the recent discovery of a new gene HBZ (HTLV-1 basic ZIP factor) encoded by the minus strand of the virus has added a new layer to the research. HBZ helps the establishment of T-cell leukemia in mice when expressed in T-lineage cells as a transgene (demonstrated by Satou et al. in 2011). HBZ seems to exert its functions by two forms – as protein and as regulatory RNA. We hypothesized that HBZ may be responsible for the accumulation in ATL precursor cells of chromosomal damages that would arrest the cell cycle progression in normal cells. We observed that HBZ-transduced mouse and human cells become resistant to apoptotic cell death or replicative senescence that is caused by clastogenic agents including arsenic trioxide. This is accompanied by the suppression of check-point mechanisms. Importantly, this function of HBZ seems mediated by the RNA form, rather than by the HBZ-protein. The nature of the RNA form of HBZ remains largely uncharacterized at the moment and our data adds new layer leading to the comprehensive understanding of how HTLV-1 causes malignant transformation of human T cells.

